# Acute adult-onset still’s disease presenting as pulmonary hemorrhage, urticaria, angioedema and leukemoid reaction: a case report and literature review

**DOI:** 10.1186/s40064-015-0924-8

**Published:** 2015-04-10

**Authors:** Sergio A Mora Alfonso, Daniel M Cuestas Rodríguez, John D Londoño, Rafael Valle-Oñate, Gerardo Quintana

**Affiliations:** Rheumatology Unit, Department of Internal Medicine, Hospital Universitario De La Samaritana E.S.E, Bogotá, Colombia; Rheumatology Unit, Department of Internal Medicine, Universidad de La Sabana, Hospital Universitario de La Samaritana E.S.E, Bogotá, Colombia; Department of Rheumatology, Universidad de La Sabana, Bogotá, Colombia; Division of Rheumatology, Department of Internal Medicine, Hospital Militar Central, Bogotá, Colombia; Division of Rheumatology, Department of Internal Medicine, Universidad Nacional de Colombia, Hospital Universitario Fundación Santa Fe, Bogotá, Colombia; Clinical Rhematology Research Fellow, Rheumatology Unit, Hospital Universitario De La Samaritana E.S.E, Cra 18A # 10 – 25 sur, Bogotá, Colombia

**Keywords:** Adult-onset still’s disease, Urticaria, Angioedema, Leukemoid reaction, Pulmonary hemorrhage

## Abstract

**Introduction:**

Adult-onset Still’s disease is a rare systemic inflammatory disorder of unknown aetiology characterized by the classic triad of persistent high spiking fevers, joint pain and a distinctive salmon-colored bumpy rash however, the multiorgan involvement can be present.

**Case description:**

A 40-year-old woman previously healthy was referred to our hospital with 7 days of high fever and generalized arthralgia, The physical exam revealed angioneurotic edema detected on soles, palms and tongue and widespread red, urticated plaques in a symmetrical distribution affecting the arms, dorsal hands, upper and lower chest and back. Followed 5 days later by fever, the patient presented dyspnea, cough and hypoxemia, the imaging studies showed unilateral consolidation and pleural effusion. The bronchoscopy with bronchoalveolar lavage and skin biopsy were consistent with neutrophilic urticarial. The hematological disorders, infections and other autoimmune diseases were excluded.

**Discussion and evaluation:**

The diagnosis of adult-onset Still’s disease can be very difficult. There are no specific tests and reliance is usually placed on a symptom complex and the well described typical rash seen in most patients. In recent years, however, other cutaneous manifestations of Adult-onset Still’s disease have been reported but these are not so well known.

**Conclusions:**

The evidence of rare manifestations is growing and the early clinical presentation of Adult-onset Still’s is extremely variable, making diagnosis difficult. For this reason, data on early clinical presentation of the disease are of interest. We reported the first case of acute Adult-onset Still’s disease with the association of pulmonary hemorrhage, urticaria and angioedema including a rare systemic manifestation as leukemoid reaction.

## Background

Adult-onset Still’s disease (AOSD) is a systemic inflammatory disorder of unknown etiology and pathogenesis with a high range of manifestations to represents a diagnostic and therapeutic challenge (Bywaters 1971). The usual clinical features are spiking fever, arthritis, macular or maculopapular rash, sore throat, myalgias, lymphadenopathy and splenomegaly (Kadar and Petrovicz 2004). The diagnosis of AOSD can be very complex because there are no specific tests and reliance is usually placed on a symptom complex and the well described typical rash seen in most patients. In recent years, the evidence of rare manifestations is growing and the early clinical presentation of AOSD is extremely variable, making diagnosis difficult. For this reason, data on early clinical presentation of the disease are of interest.

We present a rare case with many unusual manifestations. As far as we know this is the second case reported before of AOSD with an initial presentation as urticaria and angioedema (Soy 2004) and the first case of acute AOSD that reported the association with pulmonary hemorrhage and leukemoid reaction (LR). We suggest to the clinicians to keep in mind these findings and emphasize the need to consider urticaria and angioedema in the differential diagnosis of cutaneous manifestations of AOSD and recognize this new finding of pulmonary hemorrhage as another possibility in the differential diagnosis of infiltrates in acute AOSD.

## Case description

A 40-year-old woman, previously healthy, Colombian, was admitted to the Department of Rheumatology at Hospital Militar Central, with a fever of 39 C, generalised arthralgia involving the elbows, wrists, proximal and distal interphalangeal joints, metacarpophalangeal and metatarsophalangeal joints, knees and ankles, and fatigue. She had been in her usual state of good health until 2 weeks before admission and these symptoms starting shortly after the patient had a sore throat for 7 days.

**Figure 1:**
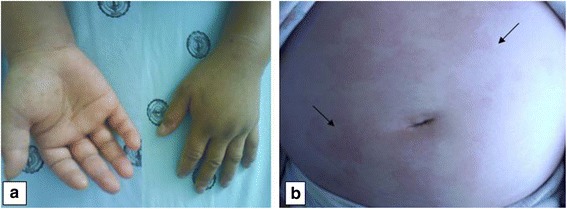
**Unusual clinical findings of AOSD in skin and hands.** A photography of dorsal and palms aspect of hands shows marked angioedema **(a)**. The urticarial eruption is evident in the abdominal photography and shows the red and urticated plaques (black arrows) **(b)**.

**Figure 2:**
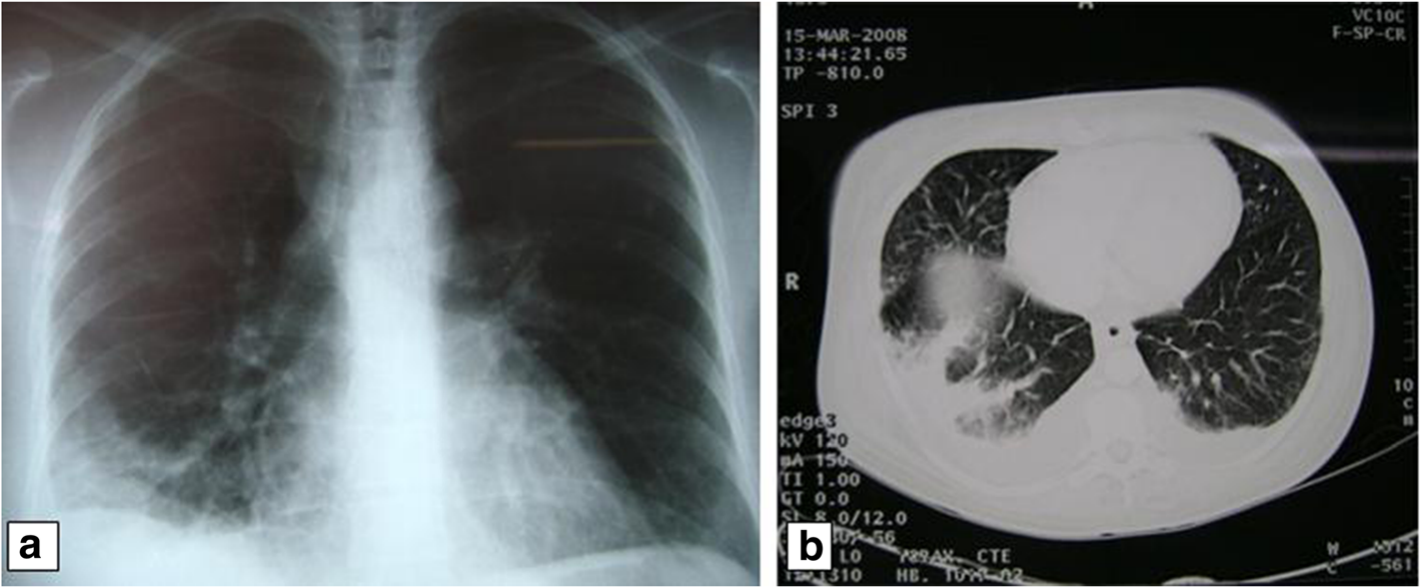
**Unusual Paraclinical findings of AOSD in lung.** A chest radiograph shows a patchy right lobe consolidation in the lower lung zone **(a)**. CT image shows extensive areas of airspace consolidation due to alveolar hemorrhage in right lung. The bilateral pleural effusions are also notable **(b)**.

Examination revealed angioneurotic edema detected on soles, palms and tongue (Figure 1a) and widespread red, urticated plaques in a symmetrical distribution affecting the arms, dorsal hands, upper and lower chest and back (Figure 1b). The rest of the examination was unremarkable. These lesions were present for less than 10 h per day and were not more pronounced at night with no associated bruising. She was diagnosed as having infection-associated-urticaria in a peripheral hospital and was treated with antibiotics (ciprofloxacin) and antihistamines (loratadine) without diminishment of her symptoms. Followed 5 days later by fever, the patient presented dyspnea, cough and hypoxemia. A chest x-ray film showed a patchy right lower lobe infiltrate (Figure 2a) and computed tomography showed unilateral consolidation and pleural effusion (Figure 2b).

**Figure 3:**
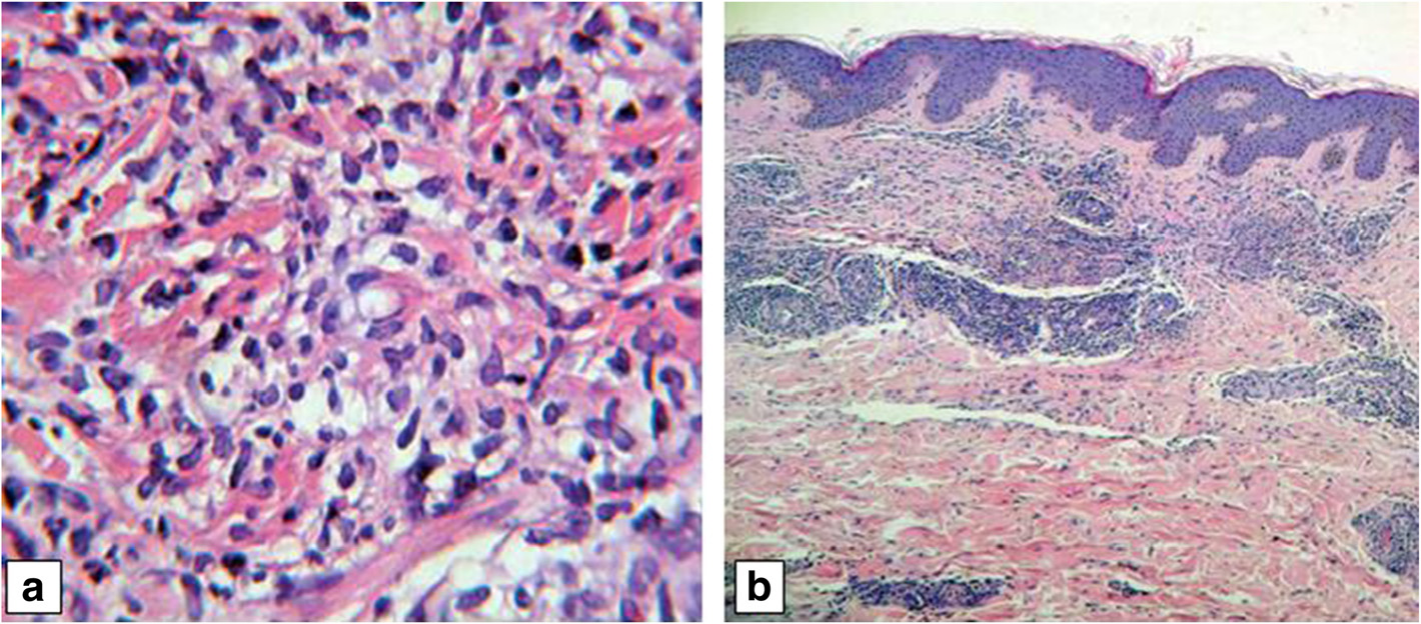
**Abdominal skin biopsy with haematoxylin-eosin-stained.** Perivascular and interstitial infiltrate composed mainly of neutrophils on upper and middle dermis with original magnification x 20 **(a)**. Shows the same specimen in more detail with original magnification x 100 **(b)**.

An early bronchoscopy with bronchoalveolar lavage was indicated revealing hemorrhagic lavage and pathological examination showed hemosiderin-containing macrophages in 80%. Bronchoalveolar lavage specimen was sent for routine bacterial cultures, fungal, viral and Pneumocystis carinii; all of the results were negative. A skin biopsy demonstrated dermal oedema, and a marked perivascular and neutrophilic infiltrate with no evidence of structural vasculitis. These features were consistent with neutrophilic urticaria (Figure 3). Other investigations revealed a normocytic anaemia Hb 11.5 g/dL erythrocyte mean corpuscolar volume 86 fl platelet count 408.000/mm^3^, a marked white blood cell count (WBC) 67.200 mm^3^ with 91% neutrophils, 5% lymphocytes, 1% eosinophils, 3% monocytes and markedly elevated acute phase reactants: erythrocyte sedimentation rate 49 mm in the first hour, C-reactive protein 11,43 mg/dl and ferritin 1650 μg/L, alanine aminotransferase was mildly elevated at 74 IU/L, lactate dehydrogenase 1200 IU/L and serum albumin reduced at 3,4 g/L. Antinuclear antibodies (immunofluorescence-Hep-2 cells), rheumatoid factor (nephelometry), antistreptolysin O, cold agglutinins, cryoglobulins and serum electrophoresis were unremarkable. There was no serological evidence for active or recent infection with the following organisms: Toxoplasma gondii, group B streptococci, hepatitis A, B or C, HIV, cytomegalovirus, tuberculosis and syphilis. Blood cultures were negative. Ultrasound investigation of the liver and biliary tract was normal. In the bone marrow aspiration, we found remarkable hyperplasia and granulocytes of each mature state, without an increase of myeloblasts, the neutrophil alkaline phosphatase score was high, Ph chromose was negative. Immunophenotyping showed mature neutrophils expressing surface antigens CD13 and CD15 at a level higher than 95% and negative for CD34 and HLA-DR.

She was diagnosed with AOSD and methylprednisolone (mini-boluses of 125 mg/ day per 3 days) was initiated as prednisone sparing therapy. The patient’s pulmonary symptoms improved with the doses of pulsed corticosteroids. After 3 days of pulsed methylprednisolone, therapy was maintained with 0.5 mg/kg of prednisone orally. Treatment with NSAIDs and prednisone controlled the rash, fever, arthritis, angioedema and significantly reduced the serum ferritin levels and all markers of activity disease. Two weeks after the pulse treatment, the chest radiography became normal. Methotrexate was prescribed for long term control of the disease at a dose of 7,5 mg/week. One month later prednisone was slowly decreased to 12,5 mg/day and withdrawn over three months. No activation period was observed during the one-year follow-up period.

## Discussion and evaluation

First described by (Bywaters 1971), AOSD has no single diagnostic test; rather, the diagnosis is based upon clinical criteria such as arthralgia, fever, skin rash, lymphadenopathy, and hepatosplenomegaly (Kadar and Petrovicz 2004). It is not uncommon for AOSD to involve other organs, such as the liver, kidney, bone marrow and, less often, the lungs. Unlike other rheumatic diseases with multiorgan involvement, in which respiratory abnormalities are well studied and documented (eg, rheumatoid arthritis, systemic lupus erythematosus, and systemic sclerosis).The pulmonary manifestations of AOSD include pleural effusion uni or bilateral (5 to 31%) or transient pulmonary infiltrates (Cheema and Quismorio 1999; Kozel and Sabroe 2004; Crispin et al. 2005), acute and chronic pneumonitis (Kadar and Petrovicz 2004), functional abnormalities and miscellaneous conditions, such as diaphragmatic dysfunction, drug-induced lung disease and respiratory distress syndrome (Cheema and Quismorio 1999). However, to the best of our knowledge, pulmonary haemorrhage in acute presentation of AOSD has not been reported before and the previous case of Sari et al (Sari et al 2009), described the first case of chronic presentation of AOSD complicated with diffuse alveolar haemorrhage during the acute flare of the disease.

The typical rash is a transient salmon-pink rash that flares during febrile episodes and is absent or minimal when the patient is afebrile. Histologic features comprised of a superficial perivascular lymphocytic infiltrate with scattered neutrophils. An eruption consisting of persistent papules and plaques has been reported frequently and is now estimated to be present in up to 65% of AOSD patients (Lee et al. 2005).This latter eruption is pruritic and often has a linear configuration, thought to be due to a Koebnerization phenomenon. However, there are an increasing number of reports of atypical cutaneous findings in AOSD, including persistent plaques and linear pigmentation, eczematous lesions, urticaria and angioedema, vesiculopustules on the hands and feet, erythema chronicum migrans, persistent generalized erythema, generalized peau d’orange-like skin infiltration (diffuse cutaneous mucinosis),acne-like lesions, non-caseating dermal granulomas, eczematous lesions and toxic eruptions (Affleck 2005).

Early diagnosis of AOSD remains a challenge to the clinicians. Physicians may not consider AOSD in the absence of the typical rash due to its apparently relative high accuracy (Masson et al 1996); however, due to the increasing number of reports of atypical cutaneous findings in AOSD with multiple morphologies, the presence of these features may be important in the diagnosis and could be incorporated into the diagnostic criteria. Urticarial rash has rarely been observed in AOSD. On reviewing the literature, Setterfield et al. (Setterfield et al. 1998) described the first case of chronic urticaria associated with AOSD presenting 9 months before the onset of the systemic signs of classic AOSD; since then many other cases have been described and including the latter, the description of urticaria in AOSD has been made in eight (8) case reports in the English literature (Affleck 2005; Setterfield et al 1998; Salaffi et al. 2009; Gesierich et al. 2009: Criado et al. 2006; Koning et al. 2007; Zendee et al. 2004) and these include the findings in five adolescent patients that indicated a disorder indistinguishable from AOSD, but not diagnosable as Systemic Juvenile Idiopathic Arthritis (SJIA) (Prendiville et al.^2004^). The presented case described both signs of urticaria and angioneurotic edema that preceded the fever and other symptoms and demonstrated histologic features of perivascular inflammatory infiltrate of the superficial dermis, composed of neutrophils and devoid vasculitis. The presence of urticaria with these histological features, can be a presenting manifestation useful in early recognition of AOSD. To the best of our knowledge, this is the second case of AOSD who presented with the combination of angioneurotic edema and urticaria in the acute phase of the disease (Soy 2004).

The evaluation of a patient with recurrent fever, urticaria and arthritis as our patient initial clinical signs should also include tests to exclude hematological, infectious, and autoimmune diseases (Table 1), eg, a complete blood count, blood cultures, serology for hepatitis, and streptococcal antibodies, tests for rheumatoid factor, antinuclear antibodies, cold agglutinins, cryoglobulins, and ferritin.

**Table 1:** **Summary of the differential diagnosis of urticaria, arthritis and fever from the literature**

**Classes of diseases**	**Diseases**
**Hereditary auto-inflammatory syndromes**	Familial cold urticaria
	Cryopyrin-associated syndrome
	Muckle–Wells syndrome
	Familial cold urticaria
	Chronic infantile neurologic cutaneous and
	articular syndrome (CINCA/NOMID)
**Infectious diseases**	Hepatitis B, C
	Chronic meningococcemia
	STAR complex (Sore Throat, Arthritis and Rash secondary to a viral infection)
	Streptococcal pharyngitis with reactive arthralgia and exanthem
**Autoimmune disorders**	AOSD
	Systemic lupus erythematosus
	Acquired C1 esterase deficiency
	Schnitzler syndrome
**Hematological disorders**	Lymphoma
	Monoclonal gammopathy of unknown significance
	Multiple myeloma
	Polyneuropathy, organomegaly, endocrinopathy, monoclonal gammopathy, and skin changes (POEMS) syndromeº
	Waldenström’s macroglobulinemia
**Other**	Hypocomplementic urticarial vasculitis
	Idiopathic chronic urticaria
	Delayed pressure urticaria
	Cryoglobulinemia
	Behçet’s disease
	Mastocytosis

Modificated and update from: (Affleck and Littlewood 2005; Koning et al. 2007; Kyle and Rajkumar 2003; Csepregi and Nemesánszky 2000; Davis and Brewer 2004; Hawkins et al. 2004; Tomkova et al. 1998; Zuberbier et al. 2009; Deacock 2008).

In AOSD common haematological abnormalities include leucocytosis, which often accompanies increased disease activity, anaemia, and thrombocytosis (Pouchot et al. 1991; Ohta et al. 1987; Wouters and van de Putte 1986). Leucocytosis is the result of a striking neutrophilia that is probably secondary to bone marrow granulocyte hyperplasia (Pouchot et al. 1991 and Min et al. 2003). In a series of 62 patients, 50% of the patients had peripheral leucocyte counts ≥ 15.000 mm^3^, and 37% had white blood cell counts ≥ 20.000 mm^3^ (Sari et al. 2009). Krumbhaar introduced the term “leukemoid reaction” in 1926 to describe leukocytic features that could mimic leukemia and that included both leukocytosis and leukopenia and it can be associated with various leukocyte types -neutrophils, lymphocytes, monocytes, eosinophils, and basophils- have been described (Krumbhaar 1926 and Sakka et al. 2006). Persistent neutrophilic leukocytosis above 50,000 cells/μL when the cause is other than leukemia defines a LR The diagnostic work-up consists of the exclusion of chronic myelogenous leukemia and chronic neutrophilic leukemia and the detection of an underlying cause as severe infections, intoxications, malignancies, severe hemorrhage, or acute hemolysis (Sakka et al. 2006). In this case the initial presentation included a LR and our initial investigations were directed to the differential diagnosis who was verified by a combination of the following: (i) a complete blood count with a peripheral blood smear that shows marked mature neutrophilia with a left shift; (ii) a high leukocyte alkaline phosphatase score; (iii) hypercellular bone marrow with intact maturation and morphology of all the elements; (iv) the absence of cytogenetic abnormalities by cytogenetic-molecular studies; (v) a mature granulocyte pattern by immunophenotyping of peripheral blood and bone marrow (Min et al. 2003).

Although LR is a rare manifestation of AOSD it has been described in the context of Fever of Uncertain Origin (FUO) (Crispin et al. 2005) and in our patient it was one of the initial diagnosis. We initially thought in AOSD after a systematic approach as we exposed before and after excluding infections, malignances and rheumatic diseases. After this work-up we applied the Yamaguichi (Yamaguchi et al. 1992) criteria and also the clinical scale designed recently by Crispin and colleagues (Crispin et al. 2005) particularly because of the initial diagnosis of FUO. Our patient met the Yamaguchi criteria for AOSD and also had a Crispin score ≥30. In support of the diagnosis of AOSD was the high serum ferritin: 1650 μg/L it remained eight times the top limit of normally during the acute phase (2 weeks). Although raised serum ferritin is not in the Yamaguchi criteria, it is regarded as a very useful tool in assisting the diagnosis of AOSD (Fautrel 2002) and ferritin levels in AOSD are usually higher than those found in patients with other autoimmune or inflammatory diseases. The validity of hyperferritinaemia as a diagnostic tool was evaluated in the retrospective study by Fautrel with 49 patients, where a fivefold increase in serum ferritin had 80% sensitivity and 41% specificity and similarly a Japanese study (Yamaguchi et al. 1992), with 82% sensitivity and 46% specificity. In this case we supported the diagnosis of AOSD based on exclusion diagnosis, clinical and paraclinical criteria.

The cause of the pulmonary hemorrhage and atypical rashes in our patient and other case reports is unclear, although proinflammatory cytokines are thought to be involved. Overproduction of interleukin (IL)-18 appears to be an important cytokine in AOSD as it is overproduced in the acute phase of the disease and with serum levels of soluble IL-2 receptors (sIL-2R) may be used as a marker for monitoring disease activity (Clio et al. 2012). Serum levels of IL1B, IL-17 and IL-6 may be potential markers for disease activity and useful for monitoring the efficacy of treatment. Otherwise, IL8 is more related to the prediction of persistent arthritis but not of disease activity. The Interferon-γ (IFN-γ) and tumor necrosis factor-α serum concentrations are elevated in AOSD patients without correlation with disease activity (Clio et al. 2012). Arndt and colleagues suggest that, in animal models endotoxemia-related lung injury was associated with increased IL-18 levels in both blood and lung tissue, and this could explain in part a possible relationship of pulmonary hemorrhage and IL-18 (Arndt et al 2000).

## Conclusions

We present a rare case of AOSD with unusual manifestations. To the best of our knowledge, this is the first case reported before of AOSD with an acute presentation as pulmonary hemorrhage, and the second case reported before as angioedema and urticaria as initial presentation. This is the only case reported the combination of these atypical features. We suggest to the clinicians to keep in mind these findings and emphasize the need to consider urticaria and angioedema in the differential diagnosis of cutaneous manifestations of AOSD and recognize this new finding of pulmonary hemorrhage as another possibility in the differential diagnosis of infiltrates in acute AOSD.

### Ethical standards and consent

This study was approved by the Institutional Ethical Committee of our Hospital; Hospital Militar Central. All techniques were performed according to the Helsinki declaration of 1975 and its modifications. Additionally, informed consent was obtained from the patient and any accompanying images for being included in the study.
